# Modification of *Escherichia coli*–bacteriophage interactions by surfactants and antibiotics *in vitro*

**DOI:** 10.1093/femsec/fiw211

**Published:** 2016-12-10

**Authors:** Pauline D. Scanlan, Anna M. Bischofberger, Alex R. Hall

**Affiliations:** 1 APC Microbiome Institute, Bioscience Building, University College Cork, Ireland; 2 Institute of Integrative Biology, ETH Zürich, 8092 Zürich, Switzerland

**Keywords:** bacteriophage, antibiotics, bile salts, *Escherichia coli*, phage therapy, genotype-by-environment interaction

## Abstract

Although experiments indicate that the abiotic environment plays an important role in bacterial interactions with their parasitic viruses (bacteriophages or phages), it is not yet clear how exposure to compounds present in nature alters the impact of phages on bacterial growth and evolution. To address this question, we exposed *Escherichia coli* K12 MG1655, in combination with three lytic phages, to various substances that natural and clinical microbial populations are likely to encounter: bile salts (present in mammalian gastrointestinal tracts), sodium dodecyl sulfate (SDS, a common surfactant in cleaning and hygiene products) and four antibiotics (present at variable concentrations in natural and clinical environments). Our results show that bile salts and SDS can reduce the detrimental effect of phages on bacterial growth. In some cases these compounds completely mitigated any negative effects of phages on bacterial growth and consequently bacteria did not evolve resistance to phages in these conditions. The proportional effects of phages were unaffected by antibiotics in most combinations, excepting three cases of phage-drug synergy. These results suggest that accounting for interactions between phages and environmental factors such as surfactants and antibiotics will improve understanding of both bacterial growth and resistance evolution to phages *in vivo* and in nature.

## Teaser

Parasitism of bacteria (*Escherichia coli*) by viruses can be prevented by compounds present in the natural environment of this species, but antibiotics do not show the same effects.

## INTRODUCTION

Bacteria–phage interactions, including those relevant to phage therapy (Sulakvelidze, Alavidze and Morris 2001; Abedon *et al.*^2011^), have been studied intensively and successfully *in vitro* (Kutter and Sulakvelidze 2004; Calendar and Abedon 2006). However, the majority of experiments have used a single set of simplified experimental conditions, which is in stark contrast to the complexity of natural microbial ecosystems. In such communities, bacteria and phages grow and evolve in chemically complex environments, making it difficult to measure the relative effects of different variables on phage infection and bacterial growth and evolution. Consequently, it is not known how environmental factors that natural and clinical populations are exposed to modify the bacteria–phage/host–parasite interactions that have been observed *in vitro.* One possibility is that compounds present outside the laboratory increase or decrease the impact of phages on bacterial population growth, and consequently the likelihood of bacteria evolving resistance to phages, compared to what we would predict from *in vitro* observations in a single set of conditions. We aimed to test this for *Escherichia coli* (strain K12 MG1655) exposed to lytic phages in a standard experimental environment (nutrient broth at 37°C) in the presence and absence of chemical compounds representing some realistic aspects of natural and clinical environments.

*Escherichia coli* is a key member of the human gut microbiota and the gut microbiota of many other warm-blooded animal hosts (Gordon and Cowling 2003). Although most strains are gut commensals, some cause common and diverse infections (Kaper, Nataro and Mobley 2004; Tenaillon *et al.*^2010^). Antibiotic resistance is a major problem in this species (World Health Organization 2014), and phage therapy has been proposed as an adjuvant or alternative solution (Brüssow 2005; Kutter *et al.*^2010^; Viertel, Ritter and Horz 2014). We hypothesized that chemicals such as bile salts, which are present in the mammalian gastrointestinal tract, can either increase or decrease the effect of phages on *E. coli* population growth observed in standard laboratory media. Bile salts are secreted into the small intestine, reaching variable final concentrations (0.2%–2% bile acid; Hofmann 1998; Gunn 2000) and promoting solubilization of fatty molecules due to detergent-like properties. Crucially, bile salts can affect a wide range of bacterial traits (Gunn 2000; Begley, Gahan and Hill 2005), some of which may be involved in interactions with phages (Baucheron *et al.*^2004^; Pumbwe *et al.*^2007^; Reen *et al.*^2012^; Hamner *et al.*^2013^). Indeed, bile salts are known to alter phage adsorption and plaque formation (Gabig *et al.*^2002^), although whether this also affects bacterial growth in the presence of phages in liquid culture or the evolution of resistance to phages is not clear.

Because bile salts are surfactants that can inhibit bacterial population growth (Begley, Gahan and Hill 2005), we also tested whether a different surfactant, sodium dodecyl sulfate (SDS), would have similar effects. This is important to determine whether effects observed for bile salts are conserved across other surfactants, but also because SDS is an ingredient in a wide range of cleaning, cosmetic and hygiene products. Bacteria and phages are therefore likely to encounter variable SDS concentrations through exposure to products containing SDS (concentrations vary widely from <3% in hygiene products such as toothpaste (Neppelberg *et al.*^2007^) to more than 30% in some cleaning and cosmetic products (Bondi *et al.*^2015^)) and in wastewater resulting from their use (Singer and Tjeerdema 1993). To determine whether observed effects were specific to surfactants or also extended to other growth-inhibiting compounds, we tested the additional hypothesis that growth inhibition by antibiotics has similar effects on bacteria–virus interactions compared to equivalent concentrations of bile salts or SDS. This hypothesis is also relevant to both phage therapy and antibiotic treatment, because phage–antibiotic interactions can potentially increase or decrease the effectiveness of antibiotic or phage+antibiotic treatments (Comeau *et al.*^2007^; Ryan *et al.*^2012^; Knezevic *et al.*^2013^; Kamal and Dennis 2015). Such interactions could come about through effects of antibiotics on cellular processes involved in phage infection or replication, or trade-offs among resistance mechanisms (Chan *et al.*^2016^; Torres-Barceló and Hochberg 2016), although these effects are likely to vary among antibiotics. We therefore included four different antibiotics.

For three different bacteriophages (representing the three Caudovirales families), we tested for non-multiplicative interactions with bile salts, SDS and four different antibiotics in terms of their effects on bacterial growth. That is, we measured the effect of each phage independently and in combination with multiple concentrations of each compound. We also measured the effect of each concentration of each compound in the absence of phages, before quantifying the deviation of bacterial growth in combination treatments from what we would expect if each phage or compound caused the same proportional change in bacterial growth as it does on its own. We employed the same statistical framework to quantify these interactions as has been used previously to infer drug–drug interactions and epistasis (Trindade *et al.*^2009^; Yeh *et al.*^2009^; Hall and MacLean 2011). We also tested whether our observations could be explained by effects of bile salts or SDS on phage survival outside the host, cation chelation or bacterial autoaggregation (Gabig *et al.*^2002^).

## MATERIALS AND METHODS

### Organisms and growth conditions

We grew *Escherichia coli* K12 MG1655 at 37°C in 100 μL Luria-Bertani broth supplemented with 10 mM MgSO_4_ and 10 mM Tris (hereafter referred to as LB) in flat-bottomed 96-well microplates in all experiments unless otherwise stated.

In experiments with bile salts, LB was supplemented with Difco Bile Salts No. 3 (Becton Dickinson, Franklin Lakes, NJ, USA). In experiments with antibiotics, we used drugs from four different classes that are associated with different resistance mechanisms (Walsh 2000; Blair *et al.*^2014^) and include some of the most widely used antibiotics as well as some of the principal classes used to treat *E. coli* infections (Johnson *et al.*^2006^; World Health Organization 2015): chloramphenicol, ciprofloxacin, streptomycin and tetracycline. In experiments below, we included each antibiotic at a range of concentrations including those that inhibit but do not prevent bacterial growth, and those that prevent growth completely.

We used three lytic phages from different families: λ (Siphoviridae), T4 (Myoviridae) and T7 (Podoviridae). The λ virus we used is an obligately lytic mutant called cI26 (Meyer *et al.*^2012^). These phages are phylogenetically and morphologically distinct, bind to different receptors [LamB (Berkane *et al.*^2006^), OmpC and LPS (Yu and Mizushima 1982), and LPS (Qimron *et al.*^2006^), respectively] and include some of the most important candidates for phage therapy of *E. coli* infections (Chibani-Chennoufi *et al.*^2004^; Brüssow 2005). We prepared stocks of each phage by picking plaques into exponentially growing 5 mL cultures of the wild-type host bacterium in LB, incubating at 37°C until lysis was visible relative to a phage-free control, then removing bacteria by adding 10% chloroform, vortexing for 1 min, centrifuging for 3 min and storing lysates at 4°C. We then diluted these stocks with LB before adding them to experimental cultures as described below.

### Growth experiments

Every growth experiment was inoculated from an independent overnight bacterial culture by 800-fold dilution, followed by 1 h of growth before addition of phages (∼500 particles of λcI26, T4 or T7 suspended in 10 μL LB or 10 μL sterile LB for phage-free treatments). For every growth experiment, we quantified the change in bacterial population density over 24 h by measuring the change in optical density (OD) at 600 nm in a M2 SpectraMax Spectrophotometer (Molecular Devices, Sunnyvale, CA, USA), calculating the growth score ΔOD, equal to OD_24h_ − OD_0h_, for each microplate well. This measure has been shown previously to detect fine-scale variation of bacterial susceptibility to viruses and viral capacity for infection (Poullain *et al.*^2008^; Hall, Scanlan and Buckling 2011). We ran eight replicate growth experiments in the presence and absence of each phage at three concentrations of bile salts, SDS and each antibiotic, plus unsupplemented LB. Bile salt and antibiotic experiments were done in the same temporal block, including the phage and no-phage treatments; SDS was tested separately in the presence of each phage alongside independent control populations (phage-free and SDS-free treatments). To avoid plate-to-plate or well-to-well variation influencing our results, we assayed the eight replicates for each treatment combination in separate microplates and in different well positions within each microplate in the main experiment; the SDS assay block was smaller and only covered two microplates, so here we included the same number of replicates for each treatment on both plates and in different well positions.

### Statistical analysis of variation in bacterial population growth

To determine whether the combined effects of phages and bile salts, SDS or antibiotics (hereafter referred to as ‘supplements’) deviated from what we would expect based on their independent effects, we compared observed scores to a null model of multiplicative effects. This is mathematically identical to a common approach for inferring epistatic interactions among genes or mutations (e.g. Phillips 2008; Trindade *et al.*^2009^; Hall and MacLean 2011), and has also been used to identify interactions among pairs of antibiotics (Yeh *et al.*^2009^). Under this model, the expected effect of a combination of a phage and a supplement at a given concentration is equal to the product of their independent effects on bacterial growth. Thus, for a given combination of phage × supplement × concentration, our null expectation is that

\begin{equation*}\Delta {\rm{O}}{{\rm{D}}_{{\rm{PS}}}} = \Delta {\rm{O}}{{\rm{D}}_{{\rm{ps}}}} \times {\rm{ }}\left( {\left( {\Delta {\rm{O}}{{\rm{D}}_{{\rm{Ps}}}}{\rm{/}}\Delta {\rm{O}}{{\rm{D}}_{{\rm{ps}}}}} \right) \times \left( {\Delta {\rm{O}}{{\rm{D}}_{{\rm{pS}}}}{\rm{/}}\Delta {\rm{O}}{{\rm{D}}_{{\rm{ps}}}}} \right)} \right),
\end{equation*}

where subscripts denote the absence and presence of phage and supplement at a given concentration (p/P for phage and s/S for supplement, with capital letters indicating presence and lowercase letters for absence). We can abbreviate this as

\begin{equation*}{W_{{\rm{PS}}}} = {W_{{\rm{pS}}}}{W_{{\rm{Ps}}}},
\end{equation*}

where *W* expresses ΔOD as a fraction of ΔOD_ps_. We tested the significance of deviations from the expected values using propagation of errors: following Silva *et al.* (2011) and Trindade *et al.* (2009), deviation from the null model (*W*_PS_ – *W*_pS_*W*_Ps_) has the error term

\begin{equation*}\sqrt {\sigma {W_{PS}}^2\ + {\rm{\ }}\sigma {W_{pS}}^2{W_{Ps}}^2 + {\rm{\ }}\sigma {W_{Ps}}^2{W_{sP}}^2},
\end{equation*}

where σ denotes the standard deviation of the replicated estimates in the corresponding phage-supplement treatment, such that greater among-replicate variance within the phage, supplement or combination treatment all increase the error term. We took deviation from the null model as significant when its squared and rooted value calculated to two decimal places exceeded the error term.

### Testing for phage resistance

In our experimental conditions, bacteria can rapidly evolve resistance to phages (Tazzyman and Hall 2015). We therefore tested whether the observed effects of bile salts and SDS on the impact of phage λcI26 on bacterial growth also altered the frequency of bacterial resistance to phages at the end of 24 h growth. We did this by testing for phage resistance in each of six replicate populations grown in the presence and absence of phage λcI26 in LB, LB + 5000 mg/L bile salts or LB + 300 mg/L SDS. We chose these concentrations because the growth experiments above indicated that they significantly alter the impact of phages. For each population, at the end of 24 h growth with or without phages, we streaked bacteria onto agar plates and incubated them overnight to obtain single, phage-free colonies. We then picked 10 colonies at random from each population and streaked them across a line of phage λcI26 (20 μL containing ∼6 × 10^4^ phages from stock lysate) that had been dried onto a LB agar plate. After overnight incubation, we followed previous studies in scoring colonies as resistant if there was no visible inhibition of bacterial growth in the segment of the streak that had been exposed to phage, and sensitive if there was inhibition (Buckling and Rainey 2002; Brockhurst *et al.*^2007^). We then took the resistance of each population as the fraction of colonies that were resistant, before plotting the resistance of individual populations from each treatment.

### Physiological basis of interactive effects

We tested some of the possible mechanisms explaining observed effects of bile salts and SDS. First, we determined whether detergents modified phage survival outside the bacterial host. To do this, we used the same conditions described for our growth experiments and added 5–10 000 phage particles to bacteria-free LB, LB+bile salts or LB+SDS, incubated for 24 h, and then counted the plaque-forming units in each population, with six replicate populations in each treatment, by dilution and plating onto lawns of *E. coli* growing in LB agar. Here we tested each of the three phages at a single concentration of bile salts and SDS, choosing concentrations that the above experiments indicated to have representative effects on the impact of phages (10 000 and 300 mg/L respectively), that is, concentrations that significantly impacted the ability of phage to reduce bacterial population growth. We then tested for differential survival of each phage in the presence of each surfactant relative to the control using *t*-tests, accepting significance only after sequential Bonferroni correction for multiple testing. Second, because previous research suggests that bacterial aggregation can increase susceptibility to phage infection (Gabig *et al.*^2002^; Kirby 2012), we tested whether bile salts or SDS altered autoaggregation in our experimental conditions by the same methods described by Gabig *et al.* (2002) and Hasman, Chakraborty and Klemm (1999): we grew three replicate 10 mL cultures of the wild type in LB or LB supplemented with bile salts or SDS, before leaving them unshaken and sampling 1 cm below the meniscus periodically, measuring OD_600_ of each sample immediately and following the rate of sedimentation by the drop in OD scores over time. We then used one-way analysis of variance (ANOVA) to test for variation of the change in OD after 158 and 331 min among treatments (bile salts, SDS or unsupplemented LB). Third, because cations, specifically Mg^2+^, are required for λcI26 infections, detergents could potentially modify phage infection by chelating cations and preventing adsorption (Gabig *et al.*^2002^). We therefore tested whether supplementation with additional MgSO_4_ (10, 20 or 40 mM added at the start of the 24 h growth experiment) restored λcI26 infection in the presence of bile salts and SDS (10 000 and 300 mg/L). We determined whether MgSO_4_ altered the effect of phage in the bile and SDS treatments by the significance of the interaction term in two-way ANOVA with MgSO_4_ concentration and phage (presence or absence) as factors.

## RESULTS

### Bile salts and SDS buffer the effects of some phages

**Figure 1.**
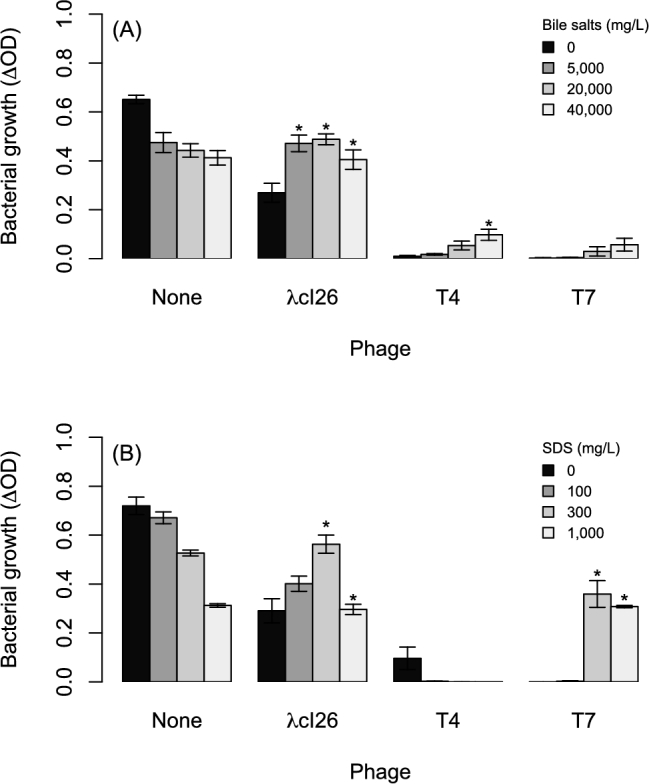
Growth of *E. coli* at various concentrations of (**A**) bile salts or (**B**) SDS in the presence and absence of three different phages. Each bar shows the average ± SE from eight replicate populations, with growth measured as the change in optical density over 24 h. Asterisks indicate values that deviate significantly from a null prediction based on the independent effects of phages and either bile salts or SDS, as described in the main text and defined by Trindade *et al.* (2009) and Silva *et al.* (2011).

In the presence of phage λcI26, *Escherichia coli* population densities were significantly greater than those predicted by our null model at all three bile salt concentrations (Fig. 1A), and not significantly different from bacterial densities observed at the same concentrations in the absence of phages (*t*-tests: *P* > 0.2 in each case). In other words, bile salts prevented phage λcI26 from reducing bacterial population growth. Although bacterial growth was also slightly higher than expected in the presence of phage T4 at the highest concentration of bile salts, bile salts had little impact on growth reduction due to phages T4 and T7 (Fig. 1A), both of which had stronger effects on bacterial growth in general compared to λcI26.

The other surfactant in our experiment, SDS, also prevented phage λcI26 from reducing bacterial population growth, which was significantly greater than that predicted by our null model at both of the two highest SDS concentrations (Fig. 1B). As observed for bile salts, at these concentrations there was no significant effect of phage λcI26 on bacterial growth (*t*-tests: *P* > 0.3 in both cases). A similar effect was observed for SDS with phage T7 (Fig. 1B), although phage action was completely prevented only at the highest concentration (*t*-test comparing with and without phage at 300 mg/L: *P* = 0.02; 1000 mg/L: *P* = 0.60). SDS did not prevent T4 from reducing bacterial growth at any of the concentrations tested (Fig. 1B).

The impact of bile salts and SDS on the reduction in bacterial growth caused by phage λcI26 was also visible in growth curves over 24 h (Fig. S1, Supporting Information) and in a separate experiment where we estimated bacterial population densities from plating and colony-forming unit counts (Fig. S2, Supporting Information).

### Antibiotics and phages interact approximately multiplicatively in most cases

**Figure 2.**
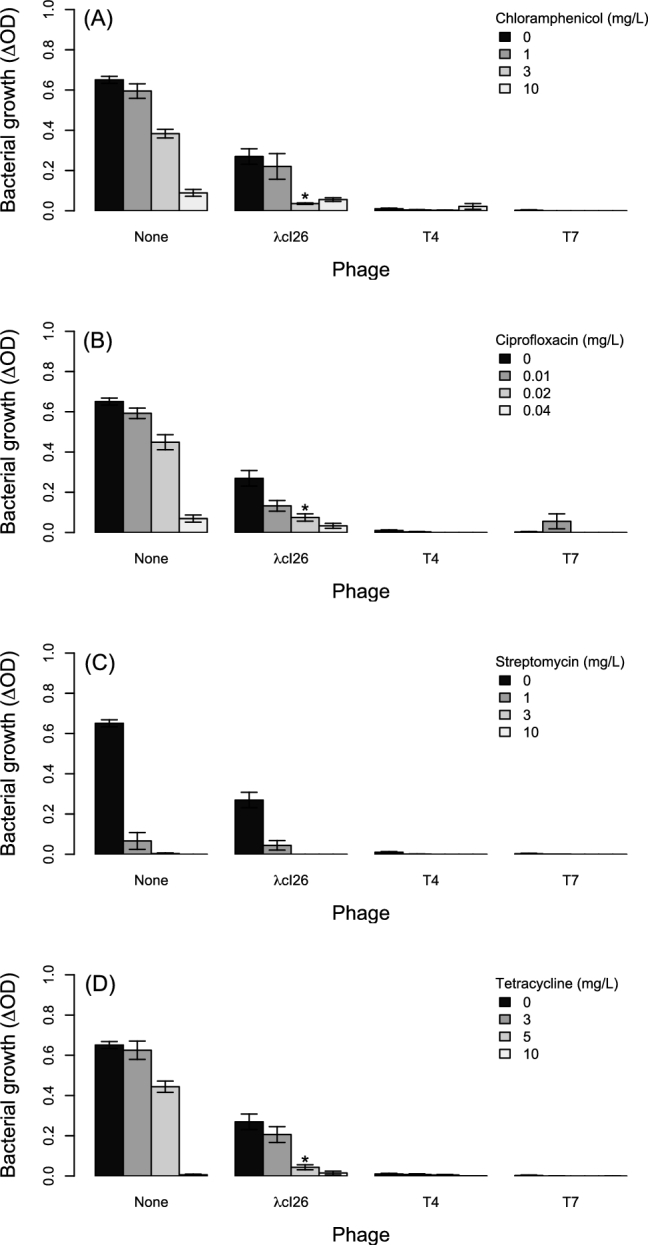
Growth of *E. coli* at various concentrations of four antibiotics: (**A**) chloramphenicol, (**B**) ciprofloxacin, (**C**) streptomycin, (**D**) tetracycline. Populations were grown in the presence and absence of all three phages (*x*-axis). Each bar shows the average ± SE from eight replicate populations. Asterisks indicate values that deviate significantly from a null prediction based on the independent effects of phages and either bile salts or SDS, as in Fig. 1.

The combined effects of antibiotics and phages on bacterial growth did not significantly deviate from that predicted by their independent effects in 33 out of 36 treatments. In the remaining three treatments, observed growth was lower than expected (3 mg/L chloramphenicol+λcI26; 0.02 mg/L ciprofloxacin+λcI26; 5 mg/L tetracycline+λcI26; Fig. 2A, B and D), although in each of these cases the deviation from our null expectation was small.

### Bile salts and SDS prevent resistance evolution

**Figure 3.**
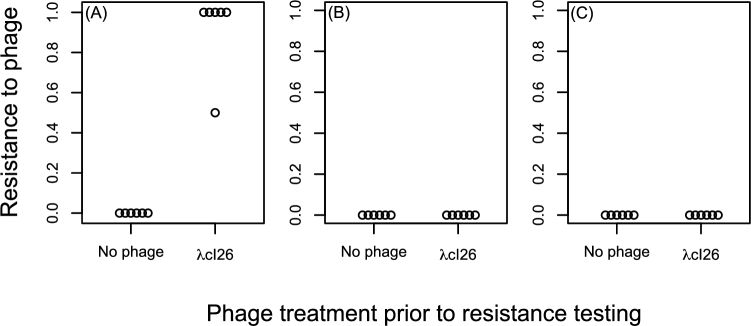
Resistance to phage λcI26 in populations grown in the presence or absence of this phage in (**A**) LB or (**B**) LB supplemented with 5000 mg/L bile salts or (**C**) 300 mg/L SDS. Each circle shows the proportion of 10 colonies in a single population that were resistant to phage λcI26, with six independent replicate populations in each treatment.

Having found that both bile salts and SDS could prevent phage λcI26 from reducing bacterial growth in liquid culture, we next tested whether this leads to a concomitant decrease in the likelihood that bacteria evolve phage resistance. We found that populations of *E. coli* that were exposed to phage λcI26 in LB consistently evolved resistance to this phage (Fig. 3A). By contrast, populations that were exposed to both phage λcI26 and either bile salts or SDS never evolved resistance to phage λcI26 (Fig. 3B and C), as observed for populations that were never exposed to phages in the same experiment.

### Extra-host survival varies among phages and environments

**Figure 4.**
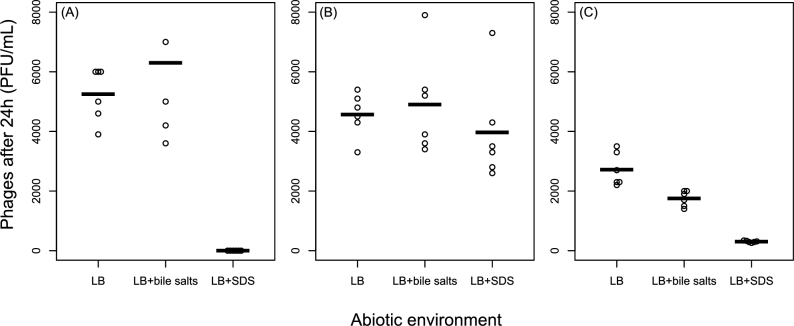
Effects of bile salts (10 000 mg/L) and SDS (300 mg/L) on phage survival outside the bacterial host for (**A**) λcI26, (**B**) T4 and (**C**) T7. Each point shows a single independent population of phages (*n* = 6 in each treatment) incubated in the same conditions and for the same duration as the main experiment but in the absence of bacteria. Bars show the mean for each treatment.

Bile salts had no effect on survival of λcI26 in the absence of bacteria (*t*-test comparing LB to LB+bile salts: *t*_10_ = 1, *P* = 0.34; Fig. 4) or T4 (*t*_10_ = 0.4, *P* = 0.67), but reduced T7 survival by 36% on average (*t*_10_ = 3.8, *P* = 0.003). SDS had no effect on T4 survival (*t*_10_ = 0.78, *P* = 0.45), but significantly reduced both λcI26 and T7 survival (Welch's *t*-tests assuming unequal variances: *t*_5_ = 14.4, *P* = 0.0001 and *t*_5_ = 10.5, *P* = 0.0001 respectively). No viable λcI26 particles were detected following treatment with SDS and T7 survival was reduced by 89% compared to unsupplemented LB on average. Thus, prevention of λcI26 and T7 infection in the presence of SDS can be at least partially explained by the effects of SDS on free phage particles. By contrast, the effect of bile salts on λcI26 infection in liquid culture does not seem to be associated with reduced survival of free phage particles.

### Detergents affect bacterial autoaggregation

**Figure 5.**
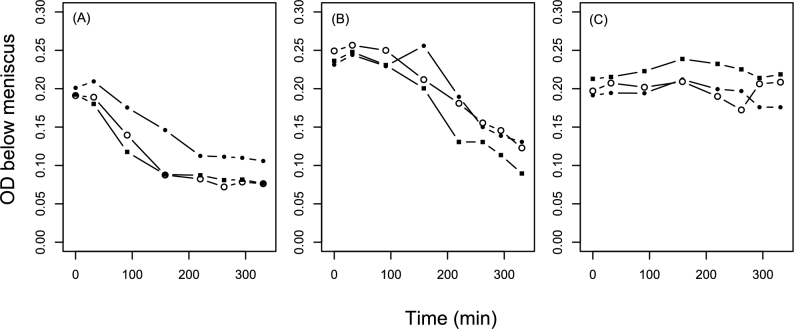
Bacterial autoaggregation in unshaken, phage-free conditions. Each series of lines and points shows an independent *E. coli* culture in (**A**) LB, (**B**) LB supplemented with 10 000 mg/L bile salts or (**C**) LB supplemented with 300 mg/L SDS. At each time point, we sampled each culture from ∼1 cm below the meniscus and measured OD immediately. Thus, rapid sedimentation leads to a rapid drop in OD shown here.

*Escherichia coli* sedimented in unshaken cultures relatively rapidly in LB, but less rapidly in LB supplemented with bile salts or SDS: OD_600_ just below the meniscus decreased relatively quickly in unsupplemented LB (Fig. 5; one-way ANOVA for variation among treatments of the change in OD after 158 min: *F*_2,6_ = 12.77, *P* = 0.007; pairwise Tukey tests show LB is significantly lower than LB+bile or LB+SDS), although by the end of the experiment the cultures in LB+bile salts had also partially sedimented (variation among treatments after 331 min: *F*_2,6_ = 47.63, *P* = 0.0002; pairwise Tukey tests show LB and LB+bile are significantly lower than LB+SDS). The relatively rapid sedimentation in LB was also observable by eye (Fig. S3, Supporting Information). Despite this, microscopy showed no clear difference in cell size, shape or spatial organization among treatments (Fig. S4, Supporting Information).

### Cation supplementation does not restore phage infection

Increasing the concentration of MgSO_4_, which is required by λcI26 for infection of *E. coli* K12 MG1655, did not restore the negative effect of this phage on bacterial growth in the presence of bile salts (MgSO_4_ concentration × phage interaction in two-way ANOVA: *F*_2,18_ = 2.69, *P* = 0.09; Fig. S5, Supporting Information) or SDS (*F*_2,18_ = 2.17, *P* = 0.14; Fig. S5), which does not support the hypothesis that Mg^2+^ chelation by bile salts causes the reduced impact of phages on bacterial growth observed in these treatments. Increasing MgSO_4_ concentration over this range (10–40 mM) did not alter the effect of phage λcI26 in the absence of bile salts either (*F*_2,18_ = 1.27, *P* = 0.31; Fig. S5).

## DISCUSSION

Natural and human-associated surfactants altered the ecological and evolutionary effects of some of the lytic phages in our experiment. Specifically, bile salts and SDS reduced the impact of phage λcI26 on *Escherichia coli* growth in liquid culture, and consequently phage-resistant mutants did not spread in these conditions. These surfactants had weaker effects on the reduction in bacterial growth caused by the other phages in our experiment. These results indicate that bile salts in the mammalian intestine, the natural environment for these species, may qualitatively change the interaction between *E. coli* and lambdoid lytic phages, although the effects of surfactants are likely to vary among different phages, as they did in our experiments. We also tested for similar interactions with four antibiotics and found that antibiotics did not modify phage effects in the same way as surfactants, ruling out the possibility that effects of surfactants were simply due to inhibition of bacterial growth. We did however find a few cases of synergy between drugs and phages, consistent with previous suggestions that phage infection can boost the efficacy of antibiotics or vice versa (Escobar-Páramo, Gougat-Barbera and Hochberg 2012; Zhang and Buckling 2012; Torres-Barceló *et al.*^2014^). On average, phage λcI26 had weaker effects on bacterial growth in unsupplemented LB compared to T4 and T7, in line with previously reported variation of adsorption and replication rates among these phages (De Paepe and Taddei 2006) and the low phages to bacteria ratio at the start of the experiment.

Our finding that bile salts reduced the impact of some phages in liquid culture is consistent with previous observations that bile salts can inhibit phage adsorption and plaque formation (Gabig *et al.*^2002^). Those authors found that sensitivity to phage λ was increased in *E. coli* expressing Ag43, a phase-variable outer membrane protein associated with increased autoaggregation and sedimentation. Consistent with this, we found that both bile salts and SDS reduced autoaggregation and sedimentation. This also supports the earlier observation (Kirby 2012) that aggregation of *Staphylococcus aureus* increases susceptibility to phage, and the notion that clusters of host cells may be relatively susceptible to phage infection (Abedon 2012).

As well as building on previous work, our results provide some novel insights: (i) bile salts can completely prevent the reduction in bacterial growth due to infection by some phages in liquid culture, thus preventing bacteria from evolving resistance to the respective phage. Impacts on bacterial population size and resistance are particularly important in the context of phage therapy where, as with antibiotics, these are key determinants of treatment outcomes (Levin and Bull 2004; Brüssow 2005). (ii) As well as bacterial autoaggregation, bile salts reduced extra-host survival of one phage (T7), indicating that interactions between phages and other substances can stem from a combination of physiological changes to both phages and bacteria. (iii) SDS had qualitatively similar effects on growth and resistance compared to bile salts, and even stronger effects on phage survival; SDS is extremely common in cleaning and hygiene products and therefore likely to be encountered in variable concentrations by bacteria and viruses outside the laboratory, including in human-associated environments such as skin and the gastrointestinal tract (Singer and Tjeerdema 1993). Our finding that SDS is deleterious to phages builds on evidence that it is an effective antiviral, as suggested by previous work with herpes simplex type 2 and HIV type 1 (Howett *et al.*^1999^), although we note that those are enveloped viruses and therefore the antiviral mechanism may be different.

Although the phage–antibiotic interactions we observed were weaker than those between bile salts or SDS and some phages, the notion that phage+antibiotic combinations can be more effective at reducing bacterial growth than we would expect from their independent effects is potentially important for both phage and antibiotic therapies. The impact of antibiotics may be modified by phages that are naturally present in humans and animals (Breitbart *et al.*^2003^; Ogilvie and Jones 2015), and combination therapy using phages and antibiotics may be more or less effective depending on these interactions (Viertel, Ritter and Horz 2014). In support, analogous interactions between pairs of antibiotics have been shown to strongly affect clearance, resistance and optimal dosing *in vitro* (Hegreness *et al.*^2008^; Peña-Miller *et al.*^2013^; Fuentes-Hernandez *et al.*^2015^). Moreover, recent work indicates that phage–antibiotic interactions are also prevalent in other bacteria–virus–antibiotic combinations (Comeau *et al.*^2007^; Escobar-Páramo, Gougat-Barbera and Hochberg 2012; Zhang and Buckling 2012; Torres-Barceló *et al.*^2014^; Kamal and Dennis 2015).

One limitation of our approach is that phage–antibiotic synergy becomes difficult to detect when the phage or antibiotic has very strong effects independently, because the expected growth score in the combination treatment is close to zero. Thus, we cannot rule out additional, undetected phage–drug synergy for some combinations. However, this would not have prevented us from detecting antagonistic interactions, as observed with bile salts and SDS. This was the main reason for including the antibiotic treatments in our growth experiments, which showed conclusively that the antagonistic interactions between surfactants and phages were not explained by general inhibition of bacterial growth. An additional limitation is that we were unable to detect any change in cell physiology or cell–cell interactions due to bile salts or SDS by microscopy. It is possible that spatial organization is disrupted during sampling and slide preparation, and therefore some differences between treatments might only be resolved using time-lapse microscopy or microfluidics, which are beyond the scope of this manuscript. Nevertheless, our observations did not show any changes, such as filamentation or chain formation that have been implicated in altered responses to phages (Kamal and Dennis 2015).

In summary, we found that surfactants can strongly influence the effect of bacteriophages on bacterial growth and the spread of resistance to bacteriophages. A key avenue for future research is determining whether the effects of bile salts or SDS we observed *in vitro* are also manifest in the mammalian gastrointestinal tract. Realistic bile salt concentrations are likely to include the range covered by our experiments (Hofmann 1998; Gunn 2000), and exposure of bacteria and phages to SDS is probably also common at variable concentrations in nature and in humans via dermal contact and oral ingestion (Singer and Tjeerdema 1993). Although physiological effects of bile salts on bacteria have been observed *in vivo* (Begley, Gahan and Hill 2005; Giraud *et al.*^2008^; De Paepe *et al.*^2011^), whether this also affects phage infection remains to be seen.

## Supplementary Material

Supplemental material Supplementary data are available at FEMSEC online.
