# Supplementary material for: Modification of Escherichia coli–bacteriophage interactions by surfactants and antibiotics in vitro
Source: FEMS Microbiol Ecol. 2016 Dec 10;93(1):fiw211. doi: 10.1093/femsec/fiw211 (PMC5091284; doi:10.1093/femsec/fiw211)

Supplementary Material

Fig. S1. Effect of phage λcI26 on bacterial density over 24h in (A) normal LB medium, (B) the same conditions supplemented with bile salts at 10,000 mg/L and (C) SDS at 300 mg/L. White points show populations grown in the absence of phages and black points show those grown in the presence of phage λcI26. Each point is the average of 12 replicate populations ±s.e., with the change in optical density at 600nm measured every 30 minutes over 24h.

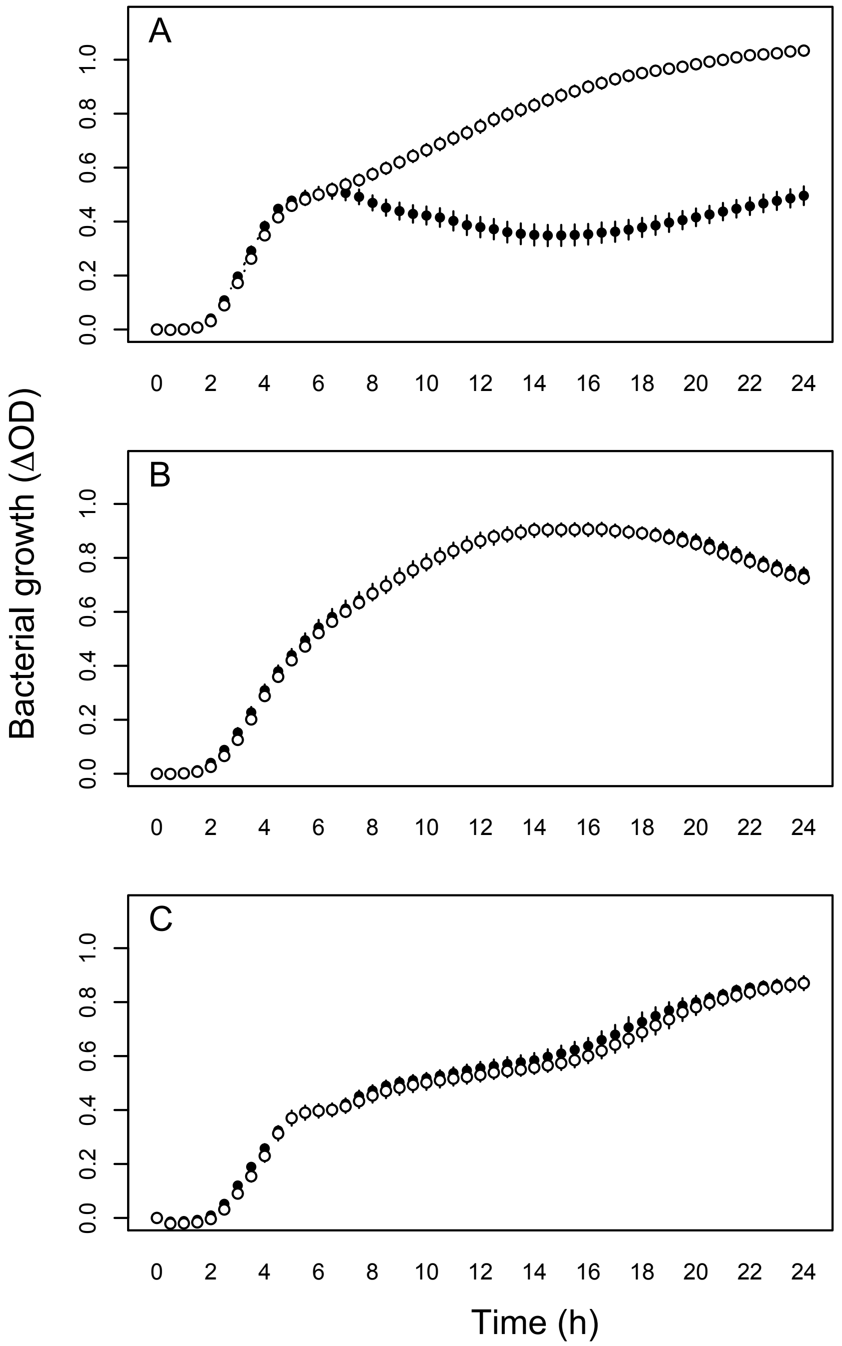

Fig. S2. Interactive effects of phages and bile salts or SDS on bacterial population growth. This experiment was carried out as in the main text (Fig. 1), except that population densities after 24 h were quantified by dilution and plating on agar, before overnight incubation and counting colony-forming units (CFUs). As in the main experiment, phage λcI26 caused a reduction in population density in (**A**) normal LB medium (Welch's $t_{4.5}$=5.82, P=0.003), but had no significant effect in the presence of (**B**) bile salts at 10,000 mg/L ($t_{4.0}$=0.33, P=0.76) or (**C**) SDS at 300 mg/L ($t_{3.7}$=2.66, P=0.06). Each circle shows a single independent population, and "X" indicates a population where no bacteria were detected at the end of the experiment.

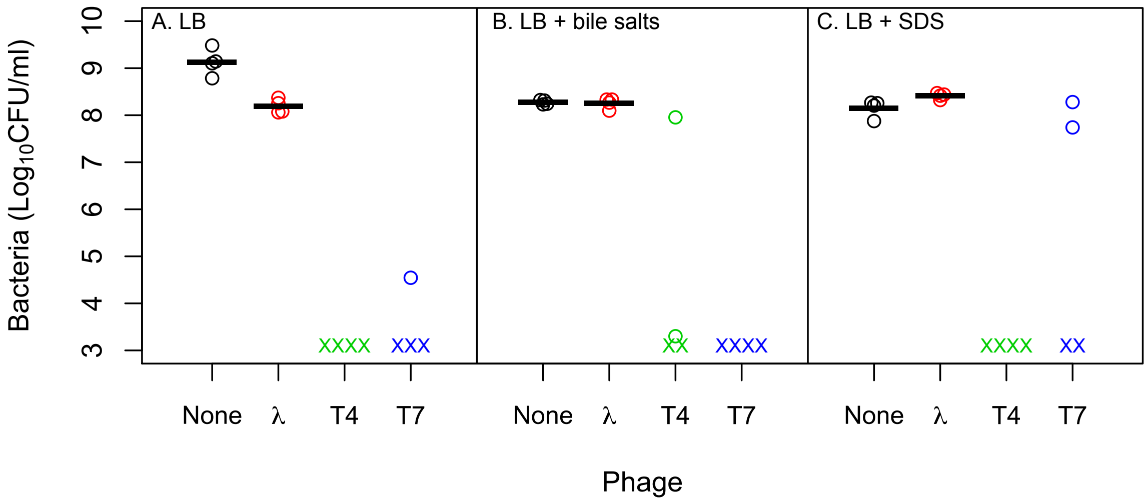

Fig. S3. Relatively rapid sedimentation in unsupplemented LB is observable by eye. Each tube was inoculated at the same time and left to stand on the bench after standardisation of OD scores. The same cultures were used for data collection for Fig. 5 in the main text.

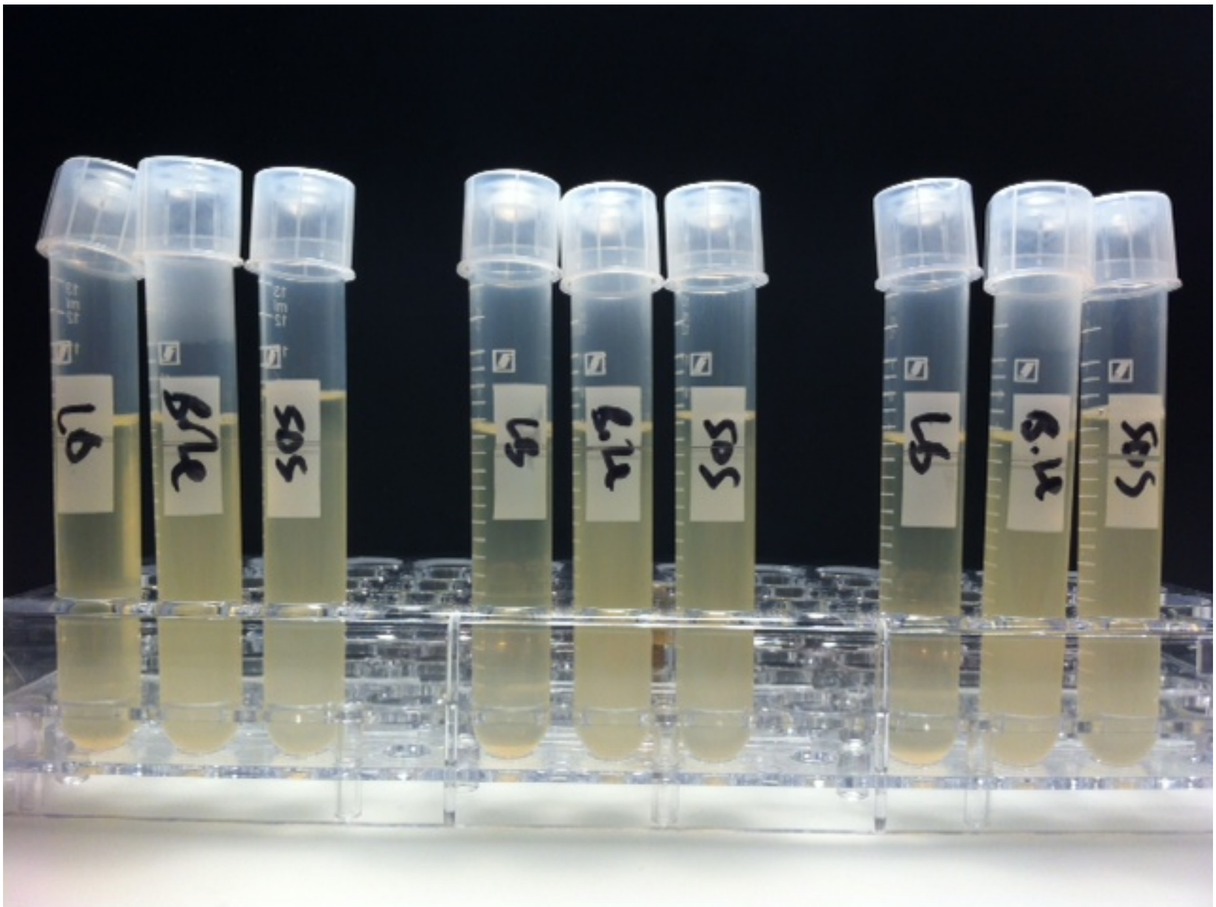

Fig. S4. Microscopic observations of *E. coli* K12 grown in LB or the same conditions supplemented with bile salts or SDS. Images were taken at 100x magnification with an Olympus IX81 microscope. Each panel is from an independent culture photographed separately. The contrast has been adjusted to make cells more visible.

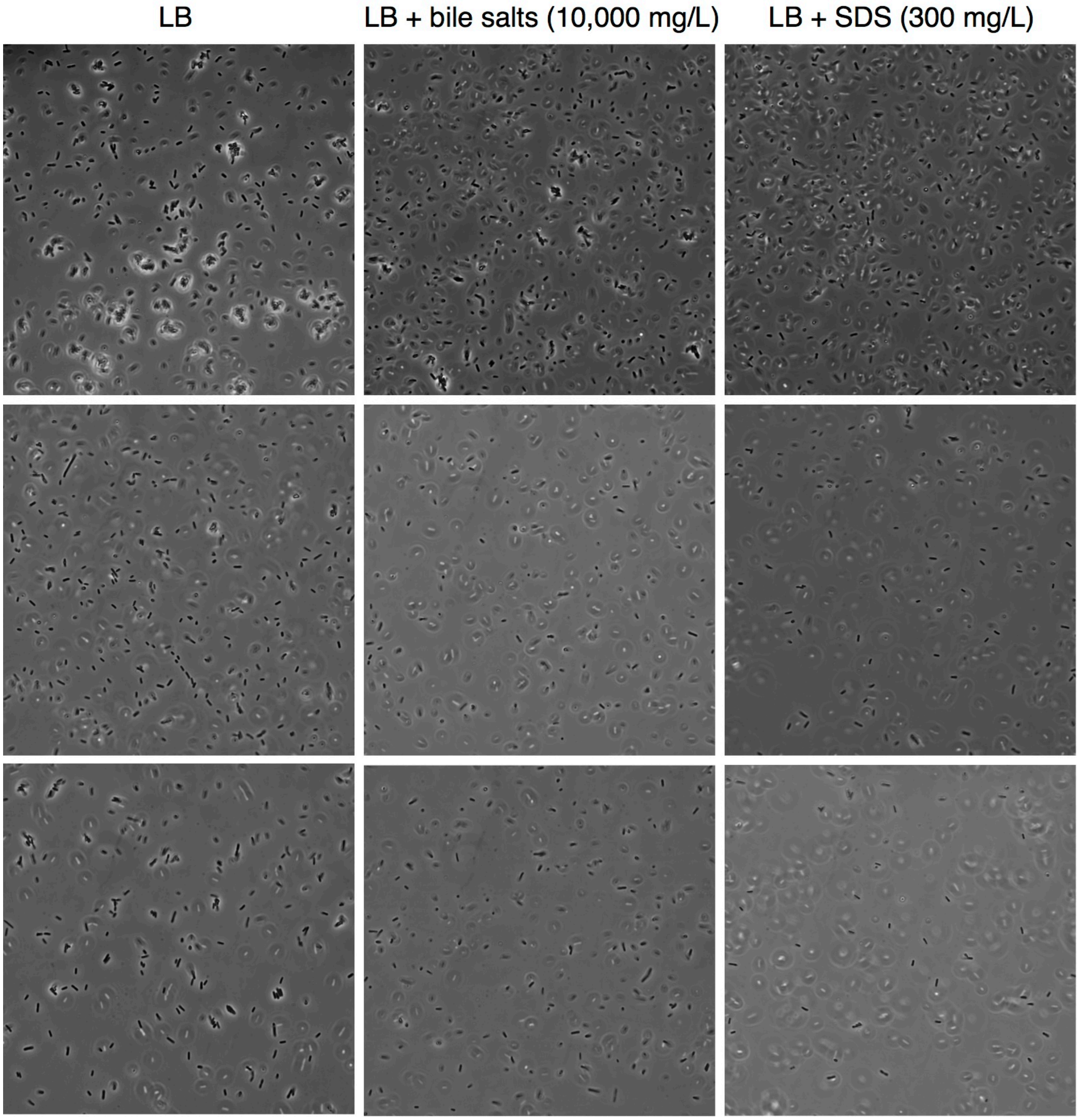

Fig. S5. The effects of bile salts and SDS on phage λcI26 infection are not alleviated by cation supplementation over the range of concentrations tested here. Each point shows the mean ±s.e. bacterial growth measured as in the main experiment, with four replicate populations grown in the presence (black circles) and absence (white circles) of phage in the same conditions as the main experiment but with variable concentrations of $MgSO_4$.

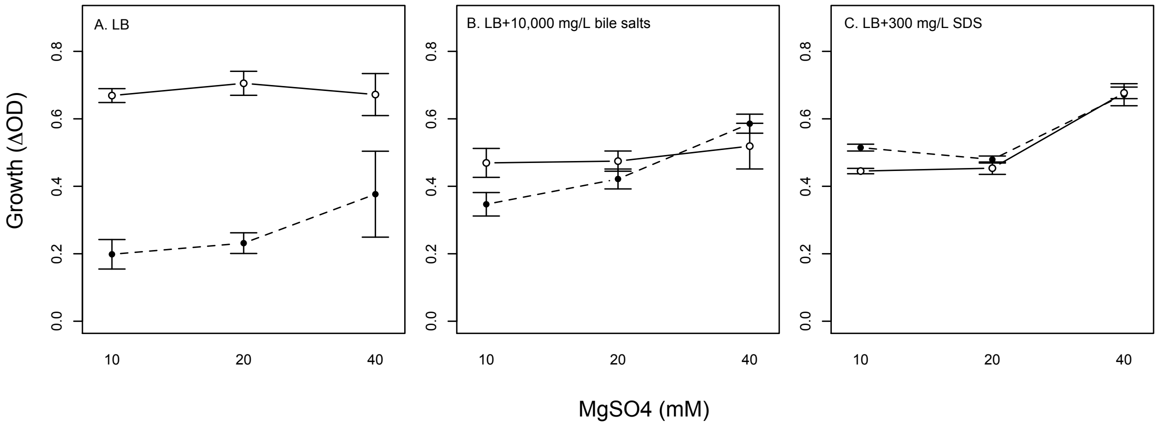

Supplement: Supplemental material — Supplementary data are available at FEMSEC online. [file fiw211_supp.pdf]
